# Tumor-Selective Cytotoxicity of Nitidine Results from Its Rapid Accumulation into Mitochondria

**DOI:** 10.1155/2017/2130594

**Published:** 2017-04-26

**Authors:** Hironori Iwasaki, Masashi Inafuku, Naoyuki Taira, Seikoh Saito, Hirosuke Oku

**Affiliations:** Tropical Biosphere Research Center, University of the Ryukyus, Nishihara, Okinawa 903-0213, Japan

## Abstract

We identified a nitidine- (NTD-) accumulating organelle and evaluated the net cytotoxicity of accumulated NTD. To evaluate tumor cell selectivity of the drug, we evaluated its selective cytotoxicity against 39 human cancer cell lines (JFCR39 panel), and the profile was compared with those of known anticancer drugs. Organelle specificity of NTD was visualized using organelle-targeted fluorescent proteins. Real-time analysis of cell growth, proliferation, and cytotoxicity was performed using the xCELLigence system. Selectivity of NTD in the JFCR39 panel was evaluated. Mitochondria-specific accumulation of NTD was observed. Real-time cytotoxicity analysis suggested that the mechanism of NTD-induced cell death is independent of the cell cycle. Short-term treatment indicated that this cytotoxicity only resulted from the accumulation of NTD into the mitochondria. The results from the JFCR39 panel indicated that NTD-mediated cytotoxicity resulted from unique mechanisms compared with those of other known anticancer drugs. These results suggested that the cytotoxicity of NTD is only induced by its accumulation in mitochondria. The drug triggered mitochondrial dysfunction in less than 2 h. Similarity analysis of the selectivity of NTD in 39 tumor cell lines strongly supported the unique tumor cell specificity of NTD. Thus, these features indicate that NTD may be a promising antitumor drug for new combination chemotherapies.

## 1. Introduction

Cancer cells express a variety of genes depending on their tissue of origin, stage, and intratumor heterogeneity [1, 2]. Drug development strategies that identify and target unique molecules expressed in specific tumor types have been attracting attention in recent years. In addition, the development of drugs to target unique characteristics of tumor cell organelles has received attention [3–5]. In previous studies, we revealed the specific accumulation of nitidine (NTD) in intracellular particles as suggestive of certain organelles [6, 7]. In addition, the degree of NTD accumulation possibly relates to the sensitivity of cell lines to the drug [6]. However, the details of accumulation and the subsequent mechanism of induction of cell death remain unclear. NTD has been known to inhibit the activity of topoisomerase-I (TOPO-I), and numerous studies have reported NTD-dependent G2/M arrest and apoptosis caused by p53 accumulation [8, 9]. A previous in vivo study showed that NTD limits neovascularization through inhibiting STAT3 and that NTD exhibits antitumorigenic effects through inhibition of vascular endothelial growth factor signaling [8]. Although several previous studies have focused on the mechanisms of cell death associated with antitumor activity of NTD, the direct target of NTD for the triggering of cell death signaling remains unclear.

In the present study, we determined the intracellular organelle into which NTD accumulates and evaluated the cytotoxicity resulting from accumulated NTD by using real-time cell proliferation analysis. In addition, we predicted the molecular targets and evaluated the action mechanisms of NTD by comparing the cell growth inhibition profiles (termed “fingerprints”) across a panel of 39 human cancer cell lines (Japanese Foundation for Cancer Research 39 (JFCR39) panel) [10]. We also compared the fingerprints of NTD with those of standard anticancer drugs using the COMPARE algorithm [10, 11].

## 2. Materials and Methods

### 2.1. Chemicals

NTD (2,3-dimethoxy-12-methyl-(1,3)-benzodioxolo(5,6-c)phenanthridinium) was used, as prepared in a previous report [6]. Camptothecin (CPT), topotecan (TPT), and paclitaxel (PTX) were purchased from Sigma-Aldrich Japan K.K. (Tokyo, Japan). JC-1 mitochondrial potential sensors were purchased from Thermo Fisher Scientific K.K. (Kanagawa, Japan). A Cell Cycle Assay Cell-Clock was purchased from Biocolor Ltd. (County Antrim, UK).

### 2.2. Cell Culture

A549 human lung adenocarcinoma cells were cultured in Dulbecco's modified Eagle's medium (DMEM) containing 10% fetal bovine serum. Cells were cultured at 37°C in a humidified atmosphere containing 5% CO_2_. Exponentially growing cells were used throughout the experiments.

### 2.3. Cell Transfection

A549 cells were transfected using an Organelle Lights intracellular targeted fluorescent proteins kit (“Peroxi-green fluorescent protein” (peroxisome), “Lysosomes-red fluorescent protein” (lysosome), “endoplasmic reticulum (ER)-RFP” (endoplasmic reticulum), “Endosomes-RFP” (endosome), “Mito-RFP” (mitochondria), and “PM-RFP” (plasma membrane)). Briefly, 10,000 A549 cells were plated into *μ*-Slide I (ibidi GmbH, Munich, Germany). After overnight preincubation for attachment, cells were treated with Organelle Lights reagent, according to the supplier's protocols. The transfected cells were treated with 5 *μ*M NTD dissolved in complete DMEM for 2 h, washed with a medium lacking the drug, and imaged using confocal laser fluorescent microscopy.

### 2.4. Staining and Analysis of the Mitochondrial Membrane Potency

A549 cells (10,000 cells/well) were incubated with 2 *μ*M JC-1 in PBS (−) for 30 min and washed twice in PBS (−). After JC-1 staining, the cells were treated with 10 *μ*M NTD, CPT, or TPT. Subsequently, the cells were observed by confocal fluorescent microscopy (0, 1, 2, 4, 8, and 21 h). All fluorescent images were analyzed and quantified using image analysis software (ImageJ). Three fixed areas were observed throughout the treatment period. The quantified fluorescent intensities are presented as relative values corrected by the intensity at 0 h.

The results are given as the mean ± standard deviation. The corrected values were compared with the control (nontreatment) group using Dunnett's test.* p* < 0.05 or* p *< 0.01 indicated statistical significance.

### 2.5. Effect of Mitochondrial Membrane Depolarization on NTD Accumulation

A549 cells (10,000 cells/well) were plated in a 96-well plate with DMEM. After 24 h, the cells were treated with 50 *μ*M carbonyl cyanide m-chlorophenyl hydrazone (CCCP) for 1 h to induce mitochondrial transmembrane depolarization. Subsequently, treatment with 5 *μ*M of NTD and 2 *μ*M of JC-1 was performed for 3 h. The fluorescence images of NTD (excitation wavelength/emission wavelength [Ex/Em] = 405/515–555 nm) and JC-1 (Ex/Em = 561/600–660 nm) were obtained by confocal laser fluorescent microscope.

### 2.6. Cell Growth and Proliferation Assay Using the xCELLigence System

A549 cells were grown and expanded in the tissue culture plates. After reaching approximately 80% confluence, cells were washed with PBS and detached from the flasks by treatment with Accutase (Innovative Cell Technologies Inc., USA). Subsequently, 50 *μ*L of cell culture media at room temperature was added to each well of an E-plate 96. Thereafter, the E-plate 96 was connected to the system and examined in the cell culture incubator for proper electrical contacts, and the background impedance was measured for 24 s. Meanwhile, the cells were resuspended in cell culture medium and the concentration was adjusted to 100,000 cells/mL. From each cell suspension, 100 *µ*L was added to each well of the E-plate 96 containing 50 *μ*L medium. After 30 min of incubation at room temperature, the E-plate 96 was placed in the cell culture incubator. Finally, the adhesion, growth, and proliferation of the cells were monitored every 10 min for up to 18 h through the incorporated sensor electrode arrays of the E-plate 96. The electrical impedance was measured by the Real-Time Cell Analyzer- (RTCA-) integrated software of the xCELLigence system as the cell index (CI), a dimensionless parameter derived to provide quantitative information regarding the biological status of the cells, such as the cell number [12].

### 2.7. Cytotoxicity Assay with Continuous Treatment Using the xCELLigence System

At 18 h after seeding during which the cells were in the log growth phase, they were exposed to 5 *μ*L of medium containing 150 *μ*M of NTD or CPT or 5 *μ*M of TPT. Controls received medium only. All experiments were conducted for 48 h. All plots were indicated as the relative values corrected by the intensity at 18 h.

### 2.8. Cytotoxicity Assay with Short-Term Treatment Using the xCELLigence System

At 18 h after seeding, the cells were exposed to 5 *μ*L of medium containing 60 or 150 *μ*M of NTD, CPT, or TPT (2 or 5 *μ*M, respectively). Controls received medium only. After 2 h of treatment, all media were replaced with complete medium. All experiments were conducted for an additional 118 h (total experiment period was 120 h). All plots were indicated as the relative values corrected by the intensity at 18 h.

### 2.9. Determination of Cell Growth Inhibition Profiles (Fingerprints) and COMPARE Analysis

Inhibition of cell growth was evaluated by the sulforhodamine B assay as described previously [10]. The concentration of NTD required for 50% growth inhibition (GI_50_) of the cells was calculated. The JFCR39 panel was used for the drug evaluation together with a database of the drug sensitivities of known compounds, including various anticancer drugs and inhibitors of biological pathways [10, 13]. The JFCR39 panel includes breast cancer, central nervous system (CNS) cancer, colon cancer, lung cancer, melanoma, ovarian cancer, renal cancer, stomach cancer, and prostate cancer cells. The graphic representation (termed fingerprint) of the mean differential growth inhibition of cells on the JFCR39 panel induced by NTD was plotted based on a calculation that uses a set of GI_50_ values. At least three independent experiments were performed, of which the representative fingerprint was used. COMPARE analysis of this fingerprint was performed by calculating Pearson's correlation coefficient (*r*) between the GI_50_ mean graphs of compounds *X* (NTD) and *Y* (known compound), using the following formula:

(1) $$\begin{array}{c} r=\frac{\left(\sum \left(x_{i}-x_{m}\right)\left(y_{i}-y_{m}\right)\right)}{{\left(\sum {\left(x_{i}-x_{m}\right)}^{2}\sum {\left(y_{i}-y_{m}\right)}^{2}\right)}^{1/2}}, \end{array}$$

where *x*_*i*_ and *y*_*i*_ are the log GI_50_ values of the compounds *X* and *Y*, respectively, for each cell line and *x*_*m*_ and *y*_*m*_ are the mean values of *x*_*i*_ and *y*_*i*_, respectively (*n* = 39) (Paull et al., 1989). The* r* values were used to determine the degree of similarity. For evaluating the fingerprint similarity between NTD and known drugs, a part of published fingerprints of drugs were obtained from previous reports about JFCR39 [14].

### 2.10. Cell Cycle Phase Quantification

A549 cells (10,000 cells/well) were plated in a 96-well plate with DMEM. After 24 h, the cells were treated with 250 nM NTD or 12 nM PTX for 48 h. Cell cycle phase quantification was evaluated by the Cell-Clock assay. For this assay, the cells were treated with a redox dye; color changes were observed 1 h after redox dye treatment and were then photographed. Observed images were analyzed by ImageJ according to Cell-Clock assay protocol.

## 3. Results

### 3.1. Organelle Localization of NTD

**Figure 1:**
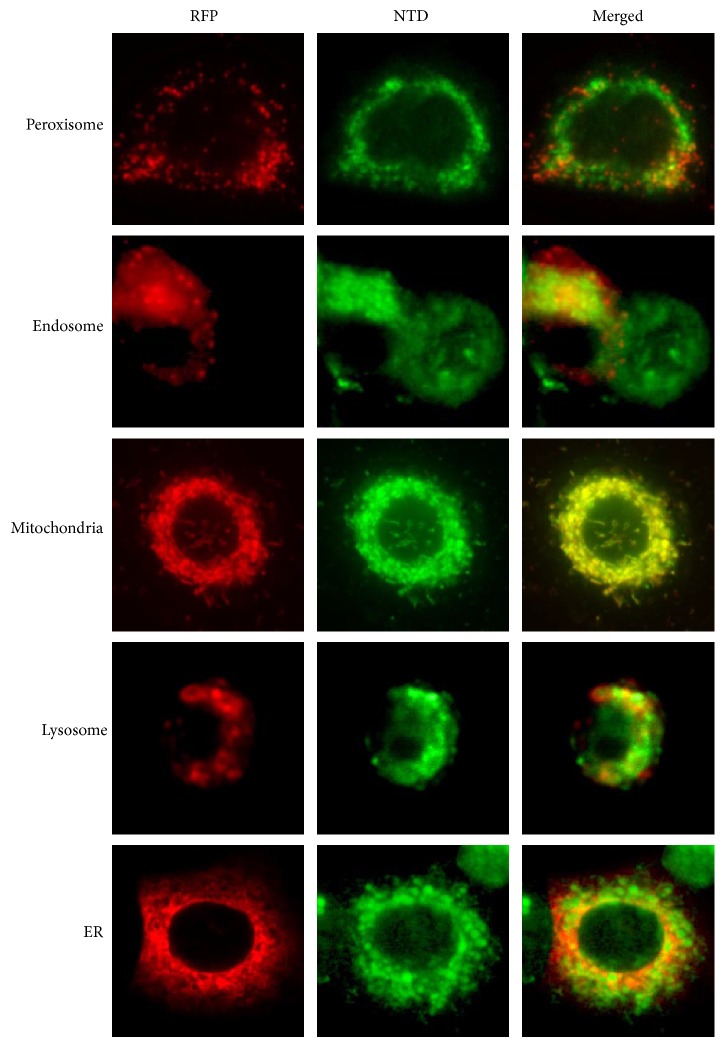
Fluorescent images of nitidine (NTD) and organelle-specific fluorescent proteins. A549 cells were transfected with each organelle-specific recombinant protein supplied in the Organelle Lights™ kit (red fluorescent protein (RFP)). The fluorescent images of NTD were observed under ultraviolet (UV) light (358 ± 28 nm).

To clarify the characteristic findings of NTD, we determined the subcellular localization of the drug in A549 cells. The intracellular organelles (endosome, lysosome, peroxisome, endoplasmic reticulum, and mitochondrion) were labeled with Organelle Lights probes. The microscopy observation indicated that NTD was enriched in the mitochondria of A549 cells (Figure 1). This result indicates that NTD localizes into mitochondria only.

### 3.2. The Change in Mitochondrial Membrane Potential Induced by NTD Accumulation

**Figure 2:**
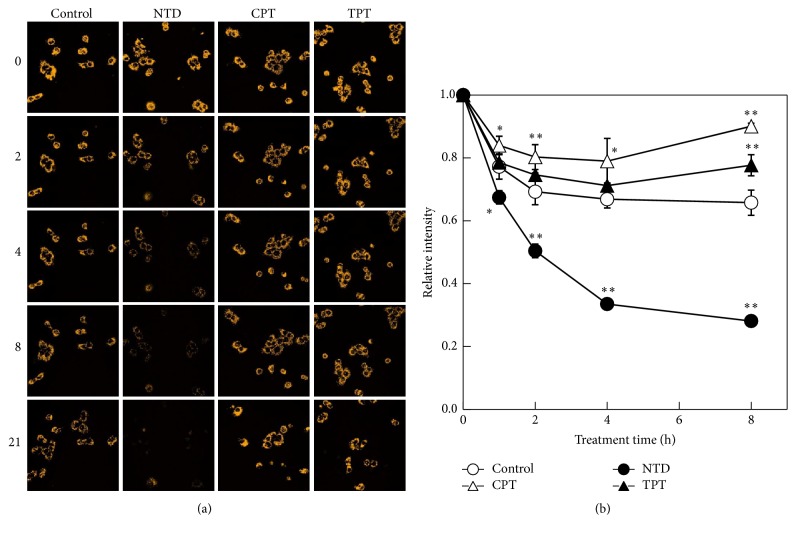
Time-dependent changes of the mitochondrial membrane potential. A549 cells were treated with JC-1 for 30 min. After washing with phosphate-buffered saline (PBS) (−), the cells were treated with nitidine (NTD), camptothecin (CPT), or topotecan (TPT) (0 h). The fluorescence of JC-1 was observed by confocal fluorescent microscopy (0, 1, 2, 4, 8, and 21 h after treatment). (a) Orange fluorescence represents JC-1, which formed J-aggregates in mitochondria in a mitochondrial membrane potential-dependent manner. (b) The fluorescent intensity of the image was quantified. All values were quantified from three random areas. Data are presented as the related intensity corrected by the value of 0 h. ^*∗∗*^*p* < 0.01; ^*∗*^*p* < 0.05.

The localization of NTD suggested that strong NTD accumulation resulted in mitochondrial dysfunction and subsequent cell death. During the next experiment, we observed changes in the mitochondrial membrane potential (MMP) of NTD-accumulating A549 cells. The MMPs of A549 cells treated with NTD, CPT, and TPT were visualized using JC-1 (Figure 2(a)). After 2 h of treatment, the JC-1 fluorescence of NTD-treated cells was slightly decreased. The apparent decrease in mitochondrial potential was observed after 4 h. After 8 h, no changes in fluorescence were observed in normal mitochondria in the NTD-treated group. The result of subquantified analysis indicated that the mitochondrial function was rapidly decreased by NTD accumulation (Figure 2(b)).

### 3.3. Effect of Mitochondrial Membrane Depolarization on NTD Accumulation

**Figure 3:**
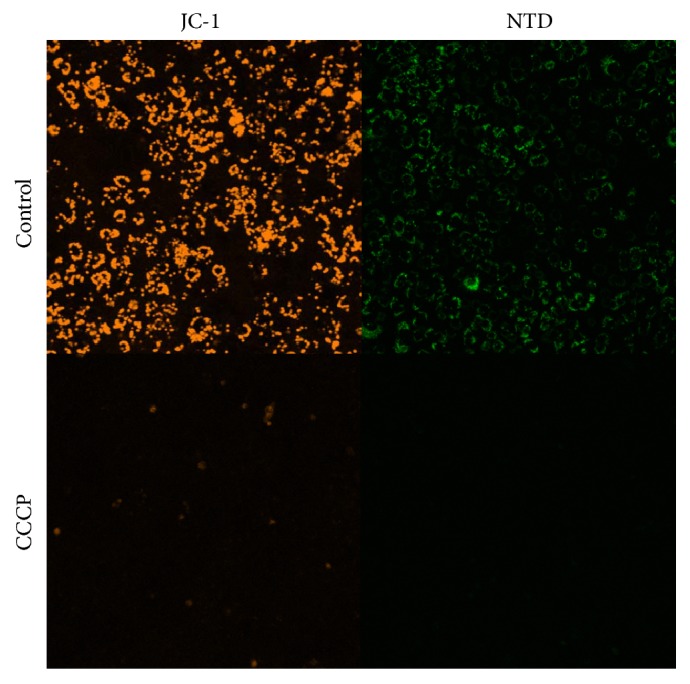
Inhibition of nitidine (NTD) accumulation into mitochondria by treatment of mitochondrial membrane depolarizer. A549 cells were treated with or without 50 *μ*M of carbonyl cyanide m-chlorophenyl hydrazone (CCCP) for 1 h. Disappearance of mitochondrial membrane potential (MMP) was detected by JC-1 (2 *μ*M) fluorescence. Control cells show accumulation of JC-1 or NTD in mitochondria. CCCP-treated cells showed disappearance of MMP (ΔΨm). In the same condition, NTD fluorescence also disappeared.

The effect of MMP on the accumulation of NTD was evaluated using MMP indicating fluorescent dye JC-1. Under the normal condition of A549 cells, JC-1 accumulated in mitochondria and formed J-aggregates with red fluorescence (colored with simulated orange images captured at 600–660 nm) (Figure 3). Under the same condition, NTD could also accumulate in mitochondria with green fluorescence (images captured at 515–555 nm). Treatment with the MMP depolarizer, CCCP, leads to the disappearance of J-aggregates of membrane potential-dependent fluorescent dye JC-1 in A549 cells. Under the same condition, the fluorescence of NTD also disappeared. This result indicated that NTD is incorporated into mitochondria depending on a high inner MMP (ΔΨm).

### 3.4. Real-Time Cell Proliferation Analysis

**Figure 4:**
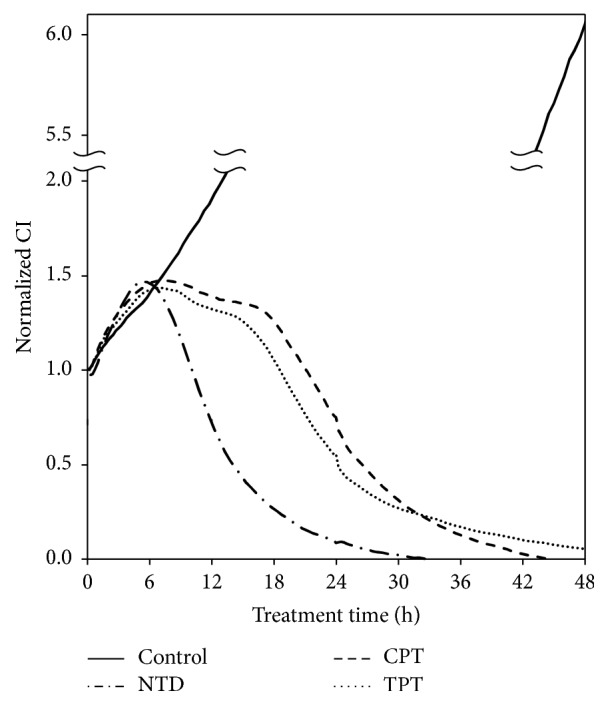
Inhibitory effect of continuous treatment with nitidine (NTD), camptothecin (CPT), or topotecan (TPT) in A549 cells measured using the xCELLigence system. Cell growth was assessed with impedance using the xCELLigence system for A549 cells. Cells were treated with 5 *μ*M of each compound after 18 h preculture (0 h). Cell growth was then monitored continuously for 48 h.

Accumulation of NTD into mitochondria depends on MMP. Under the same condition as shown in Figure 2, the MMP disappeared until 8 h after treatment of the NTD (Figure 4). These results strongly suggested that rapid cell death is inducted in A549 cells. During the next experiment, real-time cell growth analysis was performed to clarify the time-dependent manner of NTD-induced cell death. After 18 h of preincubation, A549 cells were treated with 5 *μ*M NTD, CPT, or TPT (time 0). Cell growth was monitored by measuring the electrical impedance. The growth of NTD-treated cells declined sharply after 6 h of treatment. This time-dependent curve was consistent with the relative intensity of JC-1 in A549 cells treated with NTD. The growth of CPT- or TPT-treated cells halted after 6 h and was maintained at the same range for 12 h. The decline of cells was observed after 18 h. The disappearance of MMP is not observed in Figure 2. These results suggested that this growth inhibition (6–18 h) by CPT or TPT resulted from S/G2 arrest.

### 3.5. The Effect of Short-Term NTD Treatment

In the following experiment, we evaluated the net effect of accumulated NTD in mitochondria under the same conditions as the previous experiment, excluding a change in the short-term treatment. After 2 h of treatment, the medium was replaced by a drug-free medium. Continuous NTD, CPT, and TPT treatment groups were used as references. Short-term treatment with NTD showed similar time-dependent effects as did continuous treatment. This result indicated that the rapid cytotoxicity of NTD depends strongly on the intracellular NTD concentration rather than on extracellular concentration. After short-term treatment with CPT or TPT, the growth of A549 cells was inhibited for approximately 24 h. However, surviving cells regrew after 24 h. These results indicated that NTD can accumulate rapidly in cells during various phases of the cell cycle, thereby inducing subsequent cell death. On the other hand, CPT and TPT as cell cycle arrest-inducers require extended treatment.

### 3.6. Comparison of Human Tumor Cell Line Cytotoxicity Fingerprints

The sensitivity profile of cytotoxicity of the drug to various cell lines is expected to reflect the target molecule of the drug. NTD was tested in the JFCR39 panel to evaluate its cytotoxicity profile. The GI_50_ values of NTD for each cell line in the JFCR39 panel are described in Figure 6. NTD exhibited potent growth inhibition against four cancer cell lines (CNS cancer (SNB-75), lung cancer (A549 and DMS273), and stomach cancer (MKN1)). The GI_50_ values in these cell lines were all less than half of the overall average concentration of the 39 cell lines. Among them, A549 cells were most sensitive to NTD (GI_50_ = 0.28 *μ*M). Conversely, four cell lines (CNS cancer (SF-295), colon cancer (HCT-15), and renal cancer (RXF-631L and ACHN)) were highly resistant to NTD treatment. The HCT-15 cells were especially resistant to NTD treatment. This cell line is known as inherently multidrug-resistant, expressing moderate levels of P-glycoprotein (Pgp). This result indicated that the NTD is transported by Pgp. The fingerprint of NTD was plotted based on a set of calculated GI_50_ values. The similarity of this fingerprint was compared with those of other drugs including various anticancer drugs and inhibitors of biological pathways by using Pearson's correlation coefficient (*r*) [15]. The highest* r* value was observed in PTX (tubulin binder/inhibitor) (*r* = 0.691). This* r* value (0.5 ≤* r* < 0.75) suggested that the target molecules of NTD were different from those of other compounds compared in this experiment. These results indicate that tumor selectivity of NTD was unique compared to those of existing anticancer drugs or inhibitors.

### 3.7. Comparison of the Effects of NTD and PTX on the Cell Cycle

A549 cells were treated with 250 nM NTD or 12 nM PTX for 48 h. This concentration was decided as the maximum concentration that cytotoxicity was not observed in 48 hr treatment. After treatment, the Cell-Clock assay was performed. In the Cell-Clock assay, the cell population at different stages of the cell cycle can be visualized; yellow is indicative of the G0/G1 phase, green is indicative of the S/early G2 phase, and blue is indicative of the late G2/M phase. The number of colored cells was counted by ImageJ. The change of cell cycle phases treated with each agent was evaluated as % difference compared with the nontreated cells (Figure 7). PTX treatment induced significant increase of cells in the G2/M phase. This result suggested that PTX induces G2/M phase arrest in the A549 cells. In contrast, NTD treatment induces increase in the number of cells in the G0/G1 phase and decrease in the number of cells in the S/G2 and G2/M phases. This contrasting effect on cell cycle distribution suggested that the highest* r* value observed with PTX is only simple statistical ranking.

## 4. Discussion

**Figure 5:**
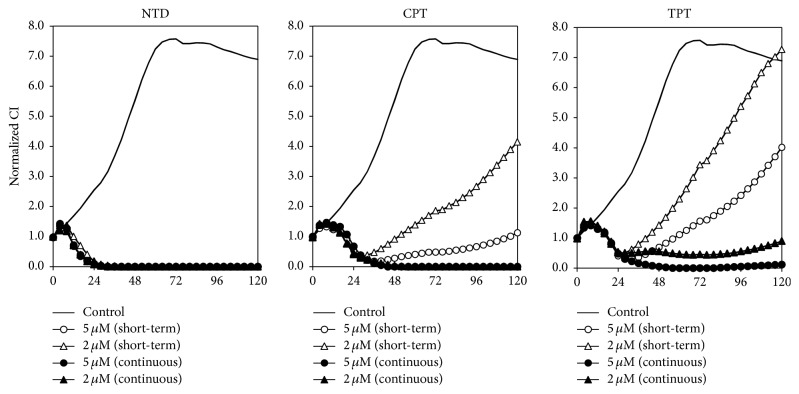
Inhibitory effect of short-term treatment with nitidine (NTD), camptothecin (CPT), or topotecan (TPT) in A549 cells measured using the xCELLigence system. Cell growth was assessed with impedance using the xCELLigence system for A549 cells. Cells were treated with 2 or 5 *μ*M of each compound after 18 h preculture (0 h). After 2 h of treatment, wells of the short-term group were washed and replaced with complete medium without compound. Cell growth was then monitored continuously for 118 h.

Benzo[c]phenanthridine derivatives such as NTD have been studied as agents showing antitumor activity stemming from their inhibition of DNA TOPO-I [16, 17]. However, NTD was not expected to display chemotherapeutic activity because of its minimal inhibition of TOPO-I. In our previous study, we suggested that NTD accumulates in intracellular organelles [7]. In addition, the potency of this accumulation correlates with IC_50_ of NTD in human lung adenocarcinoma (A549) cells [6]. In the present study, we revealed that NTD accumulates in mitochondria (Figure 1). The accumulated NTD leads to rapid disappearance of MMP in A549 cells (Figures 2(a) and 2(b)). In addition, treatment with the mitochondrial membrane depolarizer, CCCP, completely inhibited the accumulation of JC-1 and NTD (Figure 3). These results revealed that the accumulation of NTD into mitochondria is attributed to MMP, as with JC-1. Concurrently, these facts suggested that this decrease in fluorescence of JC-1 might simply be the result of competitive accumulation of NTD rather than the disappearance of MMP. However, the time-dependent proliferation curve of cell impedance in the short-term (2 h) treatment showed a similar curve as did the continuous treatment group (Figure 5). Furthermore, our previous study evaluating the clearance of intracellular NTD also showed that the intracellular concentration of NTD reached a plateau within 2 h [6]. These facts suggest that although competitive inhibition of NTD by JC-1 accumulation may occur, the major cause of the decrease of JC-1 fluorescence after 2 h in Figure 2 reflects the disappearance of MMP rather than the competitive accumulation of NTD.

NTD is one of the delocalized lipophilic cations (DLCs). DLCs are attracted by the negatively charged mitochondrial matrix and can be accumulated in the mitochondrial matrix [18, 19]. The MMP is very important for studies on tumor-selective cytotoxicity. A higher MMP has been observed in a variety of carcinomas [20]. Many recent studies suggested that a higher MMP of tumor cells compared with that of normal cells is a target for selective antitumor activity [21]. Certain DLCs have been utilized for tumor suppression in vitro and in vivo, based on this accumulation selectivity in tumor cells in response to increased MMP [21]. Most DLCs are only toxic to mitochondria at high concentrations. However, the mechanisms of mitochondrial toxicity vary (i.e., compromising the mitochondrial bioenergetic function by inhibition of adenosine triphosphatase (ATPase) [22, 23], inhibition of mitochondrial respiration through the inhibition of nicotinamide adenine dinucleotide- (NADH-) ubiquinone reductase activity in the respiratory complex I [24], or nonspecific inhibition of the membrane-binding respiratory enzymes by perturbing the mitochondrial membranes [25]). In our previous study, we did not clarify the mitochondrial toxicity. On the other hand, many previous studies have focused on DNA TOPO-I inhibition of NTD [26, 27]. To elucidate the effects of NTD accumulation and subsequent cell death, we compared the cell proliferation characteristics of NTD cells treated with CPT and TPT, known as a typical TOPO-I inhibitor. The results of real-time cell proliferation analysis showed that the time-dependent change of NTD cytotoxicity was distinctly early from CPT and TPT (Figure 4). Furthermore, the results of short-term treatment of NTD revealed that NTD-induced rapid cell death is independent of the phase of the cell cycle (Figure 5). Short-term treatment showed the same inhibitory effect as long-term treatment. The synchronization of the cell cycle was not conducted in this experiment. Consequently, the NTD accumulated in all cells independent of the phase of the cell cycle. Furthermore, these results suggested irreversible accumulation of NTD as a condition for the mitochondrial cell death to have completed by 2 h treatment. In a previous study, we observed that the accumulated NTD fluorescence does not decrease after 4 h treatment of the medium without NTD [6]. The results of the present report also support the irreversibility of NTD accumulation. These results suggested that NTD treatment might induce the mitochondrial cell death signaling pathway rather than cell cycle arrest resulting from TOPO-I inhibition. The conventional chemotherapy target tumor cells undergoing DNA replication are therefore ineffective against quiescent cells [28–30]. For these reasons, the characteristics of NTD of irreversible and rapid mitochondrial accumulation and subsequent cytotoxicity independent of the cell cycle may be useful for chemotherapy against cancer stem cells without long-term treatment, which tends to produce side effects.

The analysis of drug-resistant fingerprint similarity indicated that PTX shows a similar pattern of inhibition with a low* r* value (*r* = 0.691). Generally, an* r* value <0.75 suggests little or no similarity between the action mechanisms of two agents. In the previous report, fingerprint patterns have been indicated about representative 202 drugs at the time of publication (including various anticancer drugs and inhibitors of biological pathways) [14]. Because the fingerprints of PTX have been included in this list, we recalculated* r* value between NTD and PTX. The* r* value has been indicated to be very low (*r* = 0.42). Regarding this *r* value discrepancy, we inquired of the “Screening Committee of Anticancer Drugs (SCADS)” which is the operating organization of JFCR39. According to SCADS, “the value of the database is constantly updated and may not necessarily match the current numerical value in some cases.” These results indicated that there are plural data about PTX in JFCR39 database. In addition, it was suggested that the* r* value does not indicate consistent high value between NTD and PTX. In order to clarify the NTD-induced cell death, we further analyzed cell cycle phase change. The cell cycle analysis indicated that PTX strongly induced G2/M arrest resulting from tubulin inhibition (Figure 7). However, NTD-treated cells showed completely different cell cycle phase change. These results indicated that the mechanism of NTD-induced cell death is completely different from PTX.

If NTD is a mitochondria-target antitumor agent, it is expected that the NTD-induced cell death resulted from mitochondrial cell death. With regard to mitochondrial cell death, antimycin A is known as inhibitor of the electron transfer chain (ETC). The JFCR39 fingerprint data described above have also included the result of antimycin A [14]. The* r* value between NTD and antimycin A calculated from these data was −0.28. As a result, it was suggested that the mitochondria-specific cytotoxicity of NTD cannot be explained by only ETC inhibition. However, it is a fact that NTD accumulation into mitochondria induces the disappearance of MMP (Figure 2(a)). For elucidation of these unclear points, further research is necessary.

**Figure 6:**
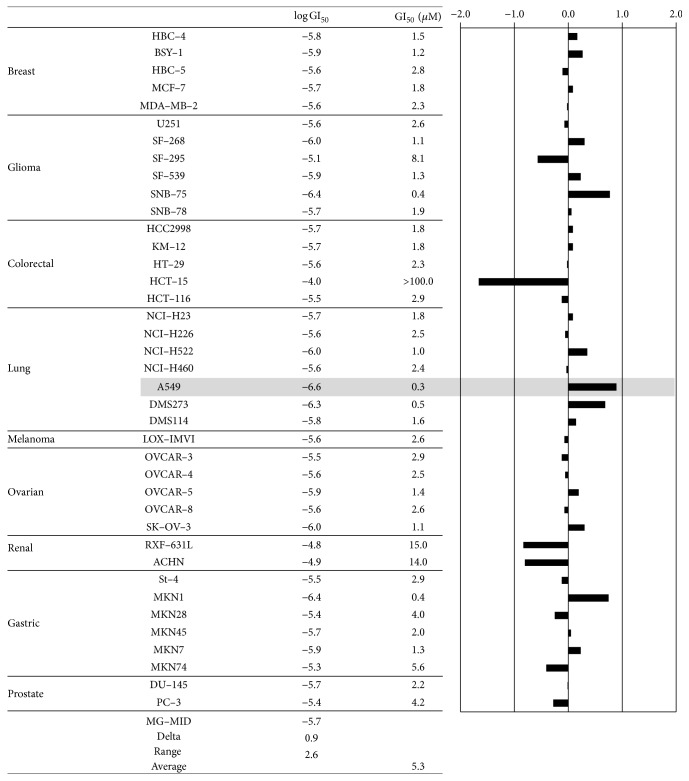
Fingerprint of NTD for the JFCR39 panel. Fingerprint indicates the differential growth inhibition pattern of NTD for the cell lines in the JFCR39 panel. The *x*-axis (solid bar) shows difference in logarithmic scale between the mean of log GI_50_ values for all 39 cell lines (MG–MID, expressed as 0 in the fingerprint) and the log GI_50_ (*μ*M) for each cell line in JFCR39 panel. Columns to the right of 0 indicate the sensitivity of the cell lines to a given compound and columns to the left indicate the resistance. MG–MID, mean of log GI_50_ values for all 39 cell lines; Delta, difference between the MG–MID and the log GI_50_ value for the most sensitive cell line; Range, difference between the log GI_50_ values for the most resistant cell line and the most sensitive cell line.

**Figure 7:**
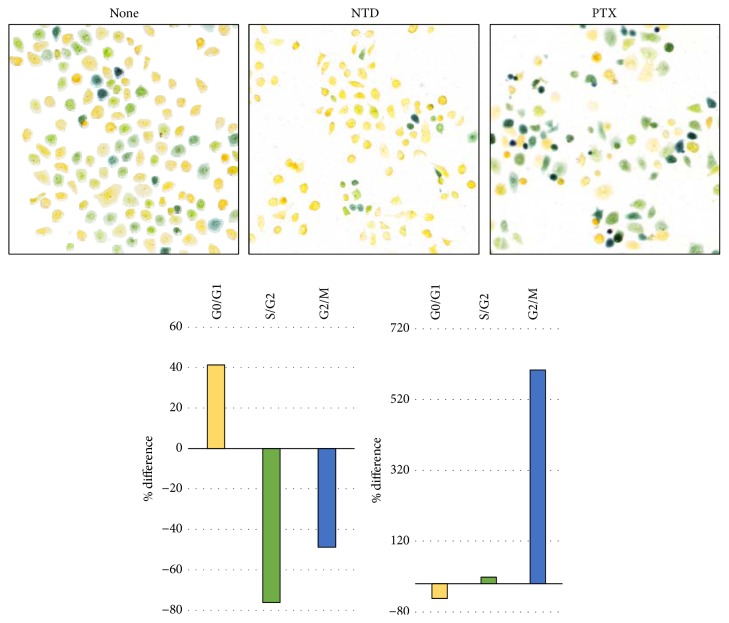
Cell cycle phase analysis. Pictures of cells indicate representative images of each treatment. Cell color changes are associated with cells in G0/G1 (yellow), S/early G2 (green), and late G2/M (blue). The histograms indicate % difference between nontreated cells and treated cells of the yellow, green, and blue cell numbers.

As a result of comparing tumor cell selectivity, we made a new observation: NTD is transported by the multidrug-resistant transporter Pgp, known as ATP-binding cassette subfamily B member 1 (ABCB1) (Figure 6). Our previous study identified ATP-binding cassette subfamily A member 1 (ABCA1) as an NTD transporter. ABCA1 is known as a cholesterol transporter [31]. These results suggested that the unique tumor cell selectivity of NTD shown in the present experiment might be supported by various features, such as the efflux activity of ABCB1, ABCA1, and MMP.

The trend toward the increasing importance of personalized medicine is growing in the field of chemotherapy. In a recent study, multidrug chemotherapy was considered as a highly important component of combination therapy. In addition, a wide variety of compounds with unique selectivity and high specificity support multidrug chemotherapy. We demonstrated certain functional features of NTD in the present study. The strategy of rapid mitochondrial accumulation of fluorescent molecules might apply not only to chemotherapy but also to in vitro diagnosis. From this perspective, NTD is considered as a useful drug for cancer chemotherapy and/or pathological analysis.
